# Integrative bioinformatics analysis of pyroptosis-related genes and immune infiltration patterns in childhood asthma

**DOI:** 10.3389/fgene.2025.1557709

**Published:** 2025-06-20

**Authors:** Di Lian, Chenye Lin, Meiling Xie, JianXing Wei, Xueling Huang, Ke Lian, Qiuyu Tang

**Affiliations:** ^1^ Pulmonology Department, Fujian Children’s Hospital (Fujian Branch of Shanghai Children’s Medical Center), College of Clinical Medicine for Obstetrics & Gynecology and Pediatrics, Fujian Medical University, Fuzhou, Fujian, China; ^2^ Department of Nephrology, Blood Purification Research Center, The First Affiliated Hospital, Fujian Medical University, Fuzhou, Fujian, China

**Keywords:** childhood asthma, pyroptosis-related genes (PRGs), immune infiltration, differentially expressed genes (DEGs), biomarkers

## Abstract

**Introduction:**

Childhood asthma (CA) is a common chronic respiratory condition that significantly impacts the respiratory function and quality of life of affected children. With a rising global incidence, CA poses substantial physical, psychological, and economic burdens. This study aimed to elucidate the role of pyroptosis-related differentially expressed genes (PRDEGs) in CA by conducting a comprehensive bioinformatics analysis using an integrated dataset from the Gene Expression Omnibus.

**Methods:**

Differential expression analysis was performed using the R package limma, identifying 2,069 differentially expressed genes (DEGs), with 1,158 upregulated and 911 downregulated genes in CA compared with the control group. Among these DEGs, 45 PRDEGs were identified, suggesting the potential involvement of pyroptosis in the pathological processes of CA. Gene Ontology and Kyoto Encyclopedia of Genes and Genomes enrichment analyses showed that PRDEGs were primarily enriched in biological processes related to the immune response, cell disassembly, and inflammatory pathways.

**Results:**

Immune cell infiltration analysis using the CIBERSORT algorithm revealed significant differences between the CA and control groups, with increased macrophages M0, activated mast cells, and γδ T cells and decreased resting natural killer cells in the CA group. Among the six hub genes identified, BAX, BECN1, MAVS, and BCL2 exhibited statistically significant expression differences between the groups (p < 0.05 in GEO data; p < 0.0001 or p < 0.001 in quantitative real-time polymerase chain reaction validation), while NOD2 and NFKBIA showed no significant differences. Receiver operating characteristic analysis of BAX, BECN1, MAVS, and BCL2 supported their potential as diagnostic biomarkers for CA, with area under the curve values ranging from 0.602 to 0.621 (95% confidence interval: 0.510–0.712).

**Discussion:**

Our findings provide novel insights into the molecular mechanisms underlying CA and highlight the diagnostic potential of BAX, BECN1, MAVS, and BCL2 as biomarkers. Targeting PRDEGs may offer new therapeutic avenues, and further research is warranted to validate these findings and explore the clinical applicability of suggested biomarkers in precision medicine for managing CA.

## 1 Introduction

Childhood asthma (CA) is a common chronic respiratory condition affecting millions of children worldwide, characterized by recurrent wheezing, shortness of breath, chest tightness, and coughing. The rising global prevalence, estimated at 10%–14% in high-income countries and over 20% in urbanized regions of developing countries, poses significant physical, psychological, and economic burdens on children, families, and healthcare systems (Asher et al., 2020; Trikamjee et al., 2022). CA is a complex, multifactorial disease influenced by genetic, environmental, and immunological factors; however, current therapeutic strategies, including pharmacological treatments and environmental management, frequently fail to adequately control symptoms or improve the quality of life in pediatric patients (Mihailidou et al., 2004; Szefler, 2007). Emerging evidence suggests a role for pyroptosis, an inflammatory form of programmed cell death, in CA pathogenesis; however, the specific involvement of pyroptosis-related genes (PRGs) remains underexplored. This study employed bioinformatics analysis to investigate the role of PRGs in CA, aiming to uncover molecular mechanisms that could inform precision medicine strategies for improved asthma care in children.

## 2 Materials and methods

### 2.1 Data download

CA datasets GSE27011 and GSE40888 were downloaded from the Gene Expression Omnibus (GEO) database (https://www.ncbi.nlm.nih.gov/geo/) using the R package GEOquery (version 2.70.0). Both datasets, derived from human blood samples, were selected due to their relevance to CA and consistency with the study’s focus on pediatric populations, with all samples from adolescents and children. The chip platform for both datasets was GPL6244, and specific information is presented in Table 1. Dataset GSE27011 comprised 36 CA cases and 18 controls, with cases stratified based on disease severity (mild to severe asthma), while GSE40888 included 65 CA and 40 controls, distinguished by allergic versus non-allergic asthma phenotypes. These datasets were selected for their sample size (totaling 101 CA cases and 58 controls), representation of diverse disease states, and compatibility with batch effect removal, enhancing the statistical power and reliability of subsequent differential expression analyses. All children in the CA and control groups were included in the study.

**TABLE 1:** Gene Expression Omnibus (GEO) microarray chip information.

Dataset	GSE27011	GSE40888
Platform	GPL6244	GPL6244
Species	*Homo sapiens*	*Homo sapiens*
Tissue	Blood	Blood
Samples in the CA group	36	65
Samples in the control group	18	40
Reference	PMID: 23222870	PMID: 25226851

GEO, gene expression omnibus; CA, childhood asthma.

PRGs were acquired from the GeneCards database (https://www.genecards.org/). The GeneCards database provides comprehensive information on human genes. Overall, 593 PRGs were identified using “Pyroptosis” as the search term and filtering for “protein-coding” PRGs. Additional PRGs were obtained in published literature from the PubMed website (https://pubmed.ncbi.nlm.nih.gov/) (Dong et al., 2021), resulting in a total of 52 PRGs. After merging and removing duplicates, 599 PRGs were obtained (Supplementary Table S1).

In this study, the R package sva (Leek et al., 2012) (version 3.50.0) was employed to eliminate batch effects from the GSE27011 and GSE40888 datasets, producing integrated GEO datasets (combined datasets) comprising 101 CA cases and 58 controls. The ComBat function within sva was utilized to adjust for systematic batch variations, ensuring data consistency between the two datasets. Principal component analysis (PCA) was conducted on the expression matrices before and after correction using the R package FactoMineR (Version 2.4) to verify the effectiveness of batch effect removal. PCA plots (Figures 2C,D) illustrated that pre-correction samples were clustered based on datasets (GSE27011 vs. GSE40888), whereas post-correction samples were clustered according to biological condition (CA vs. control), confirming the complete removal of batch effects. The combined datasets were subsequently standardized using the R package limma (Ritchie et al., 2015) (Version 3.58.1), including probe annotation, normalization, and other preprocessing steps to ensure data consistency.

**FIGURE 1:**
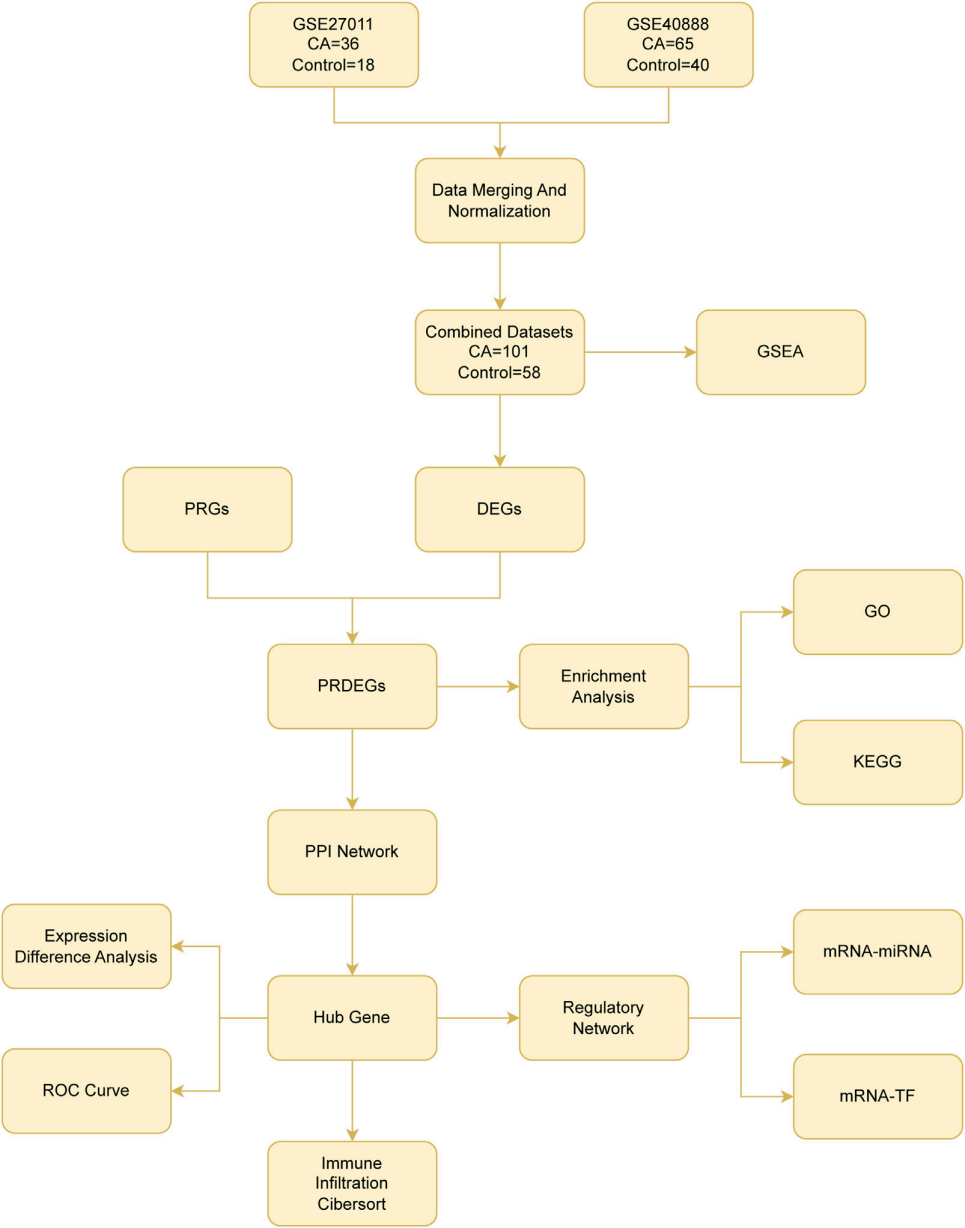
Flow chart for the comprehensive analysis of pyroptosis-related differentially expressed genes CA, childhood asthma; DEGs, differentially expressed genes; PRGs, pyroptosis-related genes; PRDEGs, pyroptosis-related differentially expressed genes; GSEA, gene set enrichment analysis; GO, gene ontology; KEGG, Kyoto Encyclopedia Of Genes And Genomes; PPI, protein–protein interaction; ROC curve, receiver operating characteristic curve; TF, transcription factor.

**FIGURE 2:**
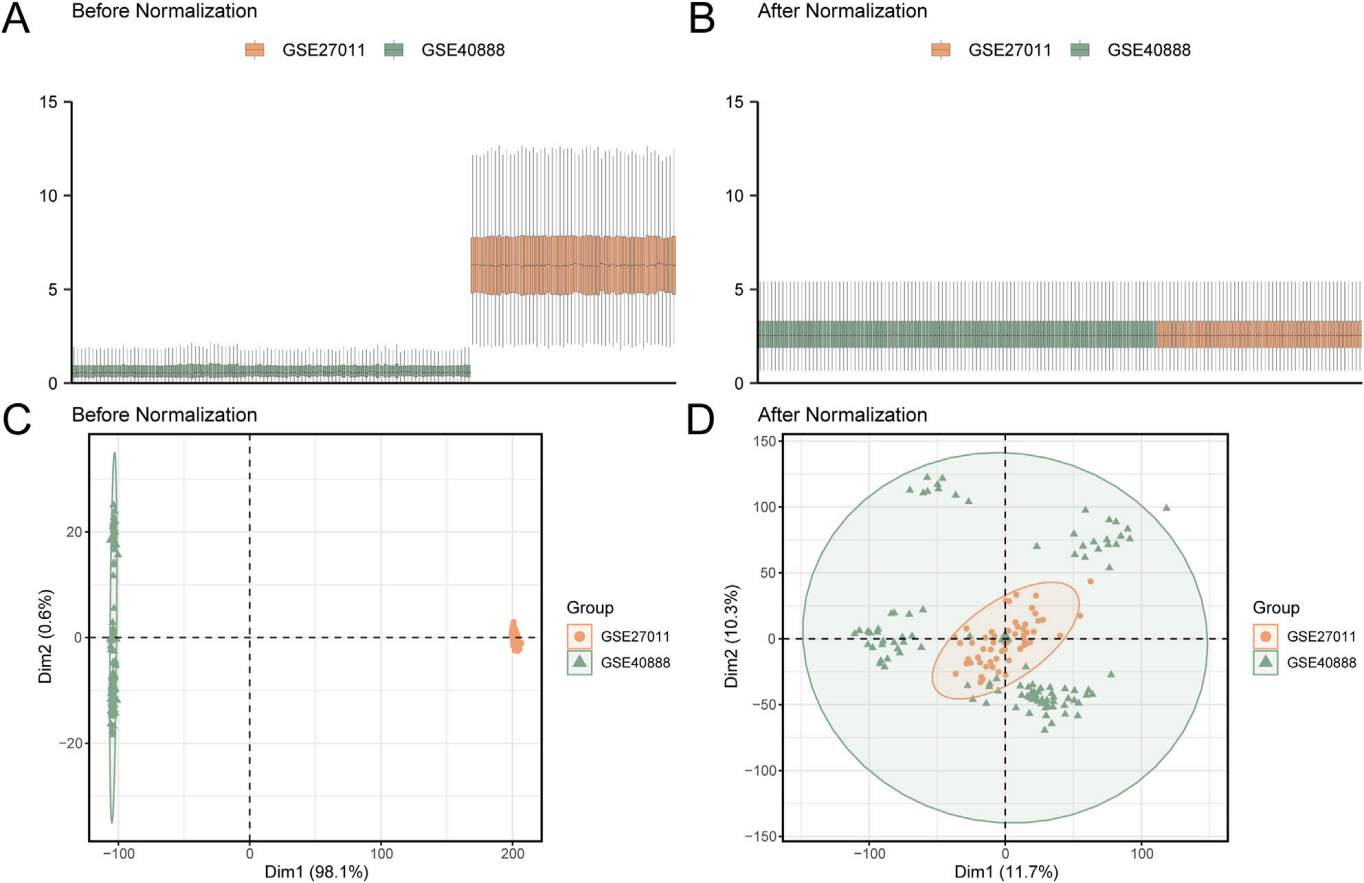
Batch effect correction of GSE27011 and GSE40888 **(A)** Boxplot of the combined GEO dataset before batch effect correction, showing significant differences in expression values and high sample variability for the CA datasets GSE27011 (orange) and GSE40888 (green). **(B)** Boxplot of the combined GEO dataset after batch effect correction, displaying a more uniform expression value distribution and reduced inter-group differences. **(C)** PCA plot of the dataset before batch effect correction, highlighting pronounced distributional differences between GSE27011 (orange) and GSE40888 (green) samples. **(D)** PCA plot of the combined GEO dataset after batch effect correction, showing increased sample clustering and overlap between GSE27011 (orange) and GSE40888 (green), indicating effective mitigation of batch effects. PCA, principal component analysis; CA, childhood asthma; GEO, Gene Expression Omnibus.

### 2.2 Differentially expressed genes (DEGs) associated with asthma-related pyroptosis in children

Based on the stratification of the combined datasets, samples were categorized into the CA and control groups. Differential expression analysis was performed using the R package limma (Version 3.58.1) with a consistent threshold set at |logFC| > 0 and p < 0.05 using the Benjamini–Hochberg (BH) method for false discovery rate (FDR) correction. Genes exhibiting |logFC| > 0 and p < 0.05 were classified as upregulated DEGs, whereas those with |logFC| < 0 and p < 0.05 were designated as downregulated. This threshold was selected to identify biologically relevant genes while mitigating multiple testing effects, thereby ensuring statistical robustness in this exploratory study. A volcano plot was generated using the R package ggplot2 (Version 3.4.4) to visualize the results, highlighting DEGs with significant changes.

To obtain pyroptosis-related DEGs (PRDEGs) associated with CA, we intersected all DEGs with |logFC| > 0 and p < 0.05 from the combined datasets with PRGs, and a Venn diagram was drawn using the R package VennDiagram (Version 1.7.3) to identify PRDEGs.

### 2.3 Gene ontology (GO) and Kyoto Encyclopedia of Genes and Genomes (KEGG) pathway enrichment analysis

GO analysis (Mi et al., 2019) is a common method used in large-scale functional enrichment studies on biological processes (BP), cellular components (CC), and molecular functions (MF). KEGG (Kanehisa and Goto, 2000) is a widely used database that stores information on genomes, biological pathways, diseases, and drugs. GO and KEGG enrichment analyses of PRDEGs were performed using the R package clusterProfiler (Yu et al., 2012) (Version 4.10.0). The entry screening criteria were adjusted to p < 0.05 (FDR or q value) < 0.25, using the BH p-value correction method.

### 2.4 Gene set enrichment analysis (GSEA)

GSEA (Subramanian et al., 2005) is employed to evaluate the distribution patterns of genes within a pre-specified gene set against a list of genes, which is organized according to its correlation with a specific phenotype, thereby elucidating its role in that phenotype. In this study, genes from the combined datasets were ranked by logFC, and analyses were performed using the R package clusterProfiler (Version 4.10.0). Parameters included 1,000 permutations, a seed value of 2,022, and gene set sizes with a range of 10–500 genes per gene set. The Molecular Signatures Database (MSigDB) was accessed for c2 gene sets (Liberzon et al., 2011). All canonical pathways were assessed with Cp. All. V2022.1. Hs. Symbols, with GMT (3050), used for GSEA. The screening criteria for GSEA were adjusted to p < 0.05, FDR value <0.25, and BH p-value correction.

### 2.5 Protein–protein interaction (PPI) network and hub gene screening

The PPI network, which represents interactions between proteins, was analyzed using the STRING database (Szklarczyk et al., 2019) to identify relationships among known and predicted proteins. PRDEGs associated with pyroptosis were investigated using STRING, with a minimum interaction score of 0.7 (low confidence) as the criterion for building the PPI network. To identify key genes, the following five algorithms from the CytoHubba plugin (Chin et al., 2014) within the Cytoscape software (Shannon et al., 2003) were employed: maximal clique centrality (MCC), degree, maximum neighborhood component (MNC), edge-percolated component (EPC), and closeness. Based on the PRDEG scores in the PPI network, the top 10 genes were selected. A Venn diagram was subsequently constructed to pinpoint overlapping hub genes across the five algorithms.

### 2.6 Construction of regulatory network

MicroRNA (miRNA) plays an important regulatory role in biological development and evolution. They regulate various target genes, while multiple miRNAs can regulate the same target gene. To analyze the relationship between pyroptosis-related hub genes and miRNAs, miRNAs related to pyroptosis-related hub genes were obtained from the starBase database (Li et al., 2014). Cytoscape software was used to visualize the mRNA-miRNA regulatory network.

Transcription factors (TFs) control gene expression by interacting with target genes (mRNAs) at the posttranscriptional stage. TFs retrieved from the hTFtarget (Zhang et al., 2020) database were merged to analyze their regulatory effects on pyroptosis-related hub genes, and the mRNA-TF regulatory network was visualized using Cytoscape software.

### 2.7 Differential expression and receiver operating characteristic (ROC) analysis of hub genes

To further explore the differences in the expression of pyroptosis-related hub genes in the CA and control groups of the combined datasets, a group comparison map was drawn based on the expression levels of pyroptosis-related hub genes. The R package pROC was applied to plot the ROC curve and calculate the area under the curve (AUC) of the pyroptosis-related hub genes. The diagnostic value of pyroptosis-related hub gene expression in CA was evaluated. The AUCs of the ROC curves were generally between 0.5 and 1. AUC >0.5 indicated that the expression of the molecule tends to promote the occurrence of the event. The closer the AUC was to 1, the better the diagnostic effect. AUC values of 0.5–0.7, 0.7–0.9, and >0.9 indicated low, moderate, and high accuracy, respectively.

### 2.8 Immune infiltration analysis

The CIBERSORT algorithm (Newman et al., 2015), based on linear support vector regression, was applied to the transcriptome expression matrix for deconvolution, enabling the estimation of immune cell composition in mixed-cell populations. Using the LM22 feature gene matrix, CIBERSORT filtered data with immune cell enrichment scores above 0 to generate an immune cell infiltration matrix for the combined datasets. Differences in immune cell infiltration between the CA and control groups were visualized using grouped comparison plots. Correlation heatmaps, created using the R package pheatmap, displayed the relationships among immune cell types and between hub genes and immune cells. Correlation coefficients (r values) were interpreted as follows: values below 0.3 indicated weak or no correlation, 0.3–0.5 represented weak correlation, 0.5–0.8 denoted moderate correlation, and above 0.8 signified strong correlation.

### 2.9 Statistical analysis

Data processing and analysis were conducted using R software (Version 4.2.2). Continuous variables were expressed as mean ± standard deviation, and comparisons between the two groups were performed using the Wilcoxon rank-sum test. In the absence of specific conditions, correlation coefficients were calculated using Spearman correlation analyses and complete result values. Statistical significance was set at P < 0.05.

### 2.10 Validation of Hub Gene Expression by quantitative real-time polymerase chain reaction (qPCR)

qPCR was performed to validate the differential expression of hub genes in peripheral blood samples from six patients with CA and six healthy controls, collected at Fujian Children’s Hospital with ethical approval (approval no: 2024 ETKLR09007). Total RNA was extracted using the TRIzol reagent (Invitrogen) and assessed for quality and concentration using a NanoDrop spectrophotometer. Complementary DNA (cDNA) was synthesized from 100 ng of total RNA using the PrimeScript RT Reagent Kit with gDNA Eraser (Takara). qPCR was conducted using the SYBR^®^ Green reagent on an Archimed R6 system with primers specific to *NOD2, NFKBIA, BAX, BCL2, BECN1*, and *MAVS* (synthesized by Sangon Biotech; sequences shown in Supplementary Table S6), as well as *GAPDH*, serving as the internal reference gene. The reaction included an initial denaturation at 95°C for 30 s, followed by 40 cycles of 95°C for 10 s, 60°C for 30 s with fluorescence acquisition, and an extension step at 95°C for 15 s and 60°C for 60 s, with a final 95°C step for 1 s to generate melting curves. Relative gene expression was calculated using the 2^-ΔΔCt method, and fold changes were analyzed in triplicate for each sample. Results were expressed as mean ± standard deviation (SD). Statistical significance was determined using the t-test on relative expression fold changes, with p < 0.05 (*), p < 0.01 (**), p < 0.001 (***), and p < 0.0001 (****) considered significant.

## 3 Results

### 3.1 Technology roadmap

The analytical workflow implemented in this study is depicted in Figure 1.

### 3.2 Merging of CA datasets

The R package sva (version 3.50.0) was utilized to eliminate batch effects from the CA datasets GSE27011 and GSE40888, generating integrated datasets. The ComBat function was used to adjust for systematic batch variations, ensuring data consistency across datasets. A distribution boxplot (Figures 2A,B) was generated to compare expression values before and after batch effect correction, revealing normalized distributions post-correction. Similarly, a PCA plot (Figures 2C,D), generated using the R package FactoMineR (Version 2.4), evaluated the distribution of low-dimensional features pre- and post-correction. These plots confirmed the effective elimination of batch effects, as pre-correction samples were clustered based on datasets (GSE27011 vs. GSE40888), whereas post-correction samples were clustered according to biological condition (CA vs. control), validating the normalization process.

### 3.3 Analysis of DEGs related to pediatric asthma-associated pyroptosis

**FIGURE 3:**
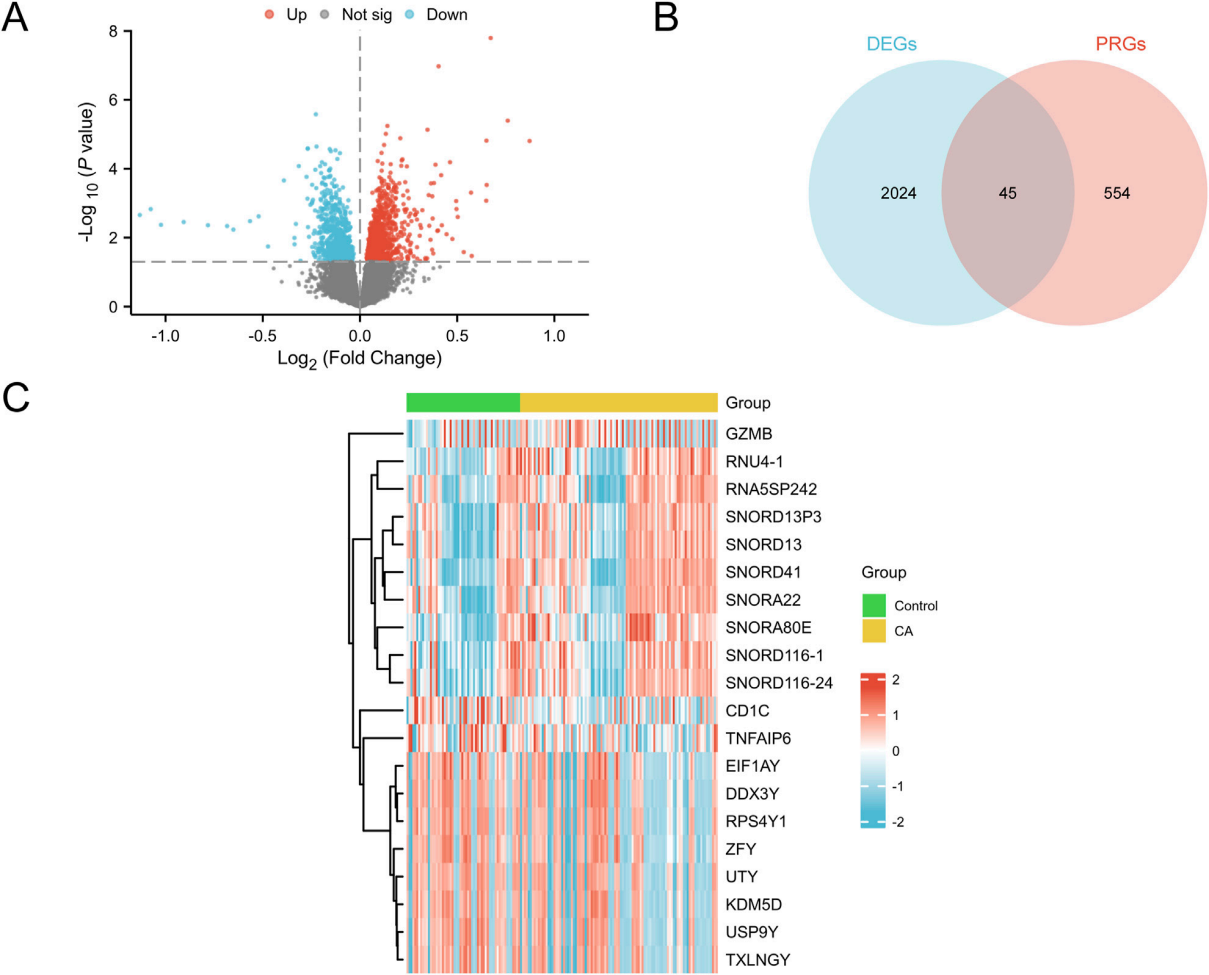
Differential gene expression analysis **(A)** Volcano plot of DEGs between the control and CA groups in the combined GEO datasets (combined datasets). Red dots represent upregulated genes (p-value <0.05 and logFC >0), blue dots indicate downregulated genes (p-value <0.05 and logFC <0), and gray dots denote genes with no significant difference; the Y-axis represents -log10 (p-value), indicating the significance of gene expression. **(B)** Venn diagram of DEGs and PRGs in the combined GEO datasets (combined datasets), illustrating the intersection between DEGs and PRGs, with 45 genes meeting both criteria. **(C)** Heatmap of the top 10 upregulated and top 10 downregulated DEGs sorted by logFC in the combined GEO datasets (combined datasets). The color in the heatmap represents gene expression levels, with red indicating high expression and blue indicating low expression; the color bar above the sample columns identifies the group, with green representing the control group and yellow representing the CA group. DEGs, differentially expressed genes; PRGs, pyroptosis-related genes; CA, childhood asthma; GEO, Gene Expression Omnibus.

Differential expression analysis using the R package limma was conducted to compare gene expression between the CA and control groups. Overall, 2,069 DEGs satisfied the criteria of |logFC| > 0 and p < 0.05, including 1,158 upregulated genes (logFC >0, p < 0.05) and 911 downregulated genes (logFC <0, p < 0.05). The results were visualized using a volcano plot (Figure 3A). PRDEGs were identified by intersecting DEGs (|logFC| > 0, p < 0.05) with cell PRGs, as shown in a Venn diagram (Figure 3B). This analysis revealed 45 PRDEGs (Supplementary Table S2). A heatmap generated using the R package pheatmap presented the top 10 upregulated and downregulated DEGs ranked by logFC (Figure 3C).

### 3.4 GO and KEGG pathway enrichment analysis of PRDEGs

**FIGURE 4:**
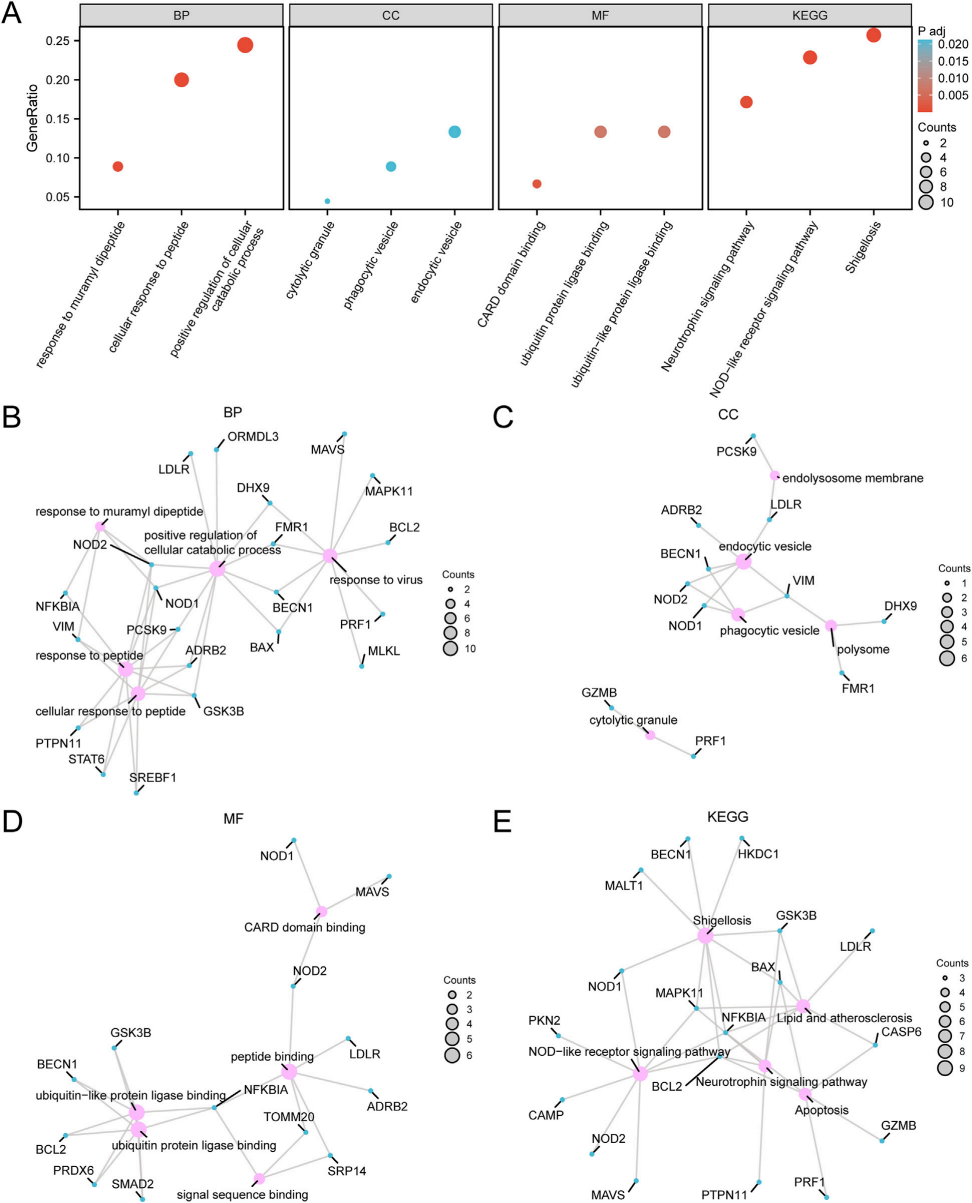
Gene ontology (GO) and Kyoto Encyclopedia of Genes and Genomes (KEGG) enrichment analysis for pyroptosis-related differentially expressed genes (PRDEGs) **(A)** Bubble chart displaying the GO and KEGG enrichment analysis results for PRDEGs, including BP, CC, MF, and biological pathways (KEGG). The x-axis represents GO terms and KEGG terms, bubble size indicates the number of genes, and bubble color reflects the adjusted p-value (adj.p-value), with darker red colors indicating smaller adj.p-values and darker blue colors indicating larger adj.p-values; the screening criteria were adj.p < 0.05 and FDR (q-value) < 0.25, with the p-value correction method being Benjamini–Hochberg (BH). **(B)** Network diagram of the GO enrichment analysis results for PRDEGs, illustrating associated terms and related molecules within the BP category, with lines indicating relationships between terms and molecules. **(C)** Network diagram of the CC enrichment analysis results for PRDEGs. **(D)** Network diagram of the MF enrichment analysis results for PRDEGs. **(E)** Network diagram of the KEGG enrichment analysis results for PRDEGs, where pink nodes represent terms, blue nodes represent molecules, lines indicate relationships between terms and molecules, and larger nodes signify a greater number of associated molecules. PRDEGs, pyroptosis-related differentially expressed genes; GO, Gene Ontology; KEGG, Kyoto Encyclopedia of Genes and Genomes; BP, Biological Process; CC, Cellular Component; MF, Molecular Function.

GO and KEGG pathway enrichment analyses were conducted to explore the relationships among BP, CC, MF, and biological pathways of the 45 PRDEGs associated with CA. The enrichment results are presented in Table 2. The results showed that the PRDEGs were primarily enriched in positive regulation of cellular catabolic processes, responses to muramyl dipeptides, cellular responses to peptides, and responses to viral infection (BP). Enriched CC pathways included endocytic vesicles, phagocytic vesicles, cytolytic granules, polysomes, and endolysosome membranes. MFs were enriched for Caspase Activation and Recruitment Domain, ubiquitin-protein ligase, ubiquitin-like protein ligase, peptide, and signal sequence bindings. KEGG pathway analysis further identified associations between pathways, including shigellosis, NOD-like receptor signaling, neurotrophin signaling, apoptosis, and lipid and atherosclerosis pathways. Results were visualized using bubble plots (Figure 4A). Additionally, network diagrams were constructed for each category (BP, CC, MF, and KEGG) based on enrichment analyses (Figures 4B–E).

**TABLE 2:** Results of gene ontology (GO) and Kyoto Encyclopedia of Genes and Genomes (KEGG) enrichment analyses for pyroptosis-related differentially expressed genes (PRDEGs).

Ontology	ID	Description	Gene ratio	Bg ratio	p-value	p-adjust	q-value
BP	GO: 0031331	Positive regulation of cellular catabolic process	11/45	449/18,800	6.23 e-09	1.37 e-05	8.26 e-06
BP	GO: 0032495	Response to muramyl dipeptide	4/45	20/18,800	1.35 e-07	1.04 e-04	6.27 e-05
BP	GO: 1901653	Cellular response to peptide	9/45	361/18,800	1.55 e-07	1.04 e-04	6.27 e-05
BP	GO: 1901652	Response to peptide	10/45	491/18,800	1.89 e-07	1.04 e-04	6.27 e-05
BP	GO: 0009615	Response to virus	9/45	392/18,800	3.10 e-07	1.37 e-04	8.22 e-05
CC	GO: 0030139	Endocytic vesicle	6/45	342/19,594	1.24 e-04	2.13 e-02	1.61 e-02
CC	GO: 0045335	Phagocytic vesicle	4/45	138/19,594	2.81 e-04	2.13 e-02	1.61 e-02
CC	GO: 0044194	Cytolytic granule	2/45	13/19,594	3.96 e-04	2.13 e-02	1.61 e-02
CC	GO: 0005844	Polysome	3/45	65/19,594	4.48 e-04	2.13 e-02	1.61 e-02
CC	GO: 0036020	Endolysosome membrane	2/45	19/19,594	8.60 e-04	3.27 e-02	2.48 e-02
MF	GO: 0050700	CARD domain binding	3/45	16/18,410	7.47 e-06	1.67 e-03	1.15 e-03
MF	GO: 0031625	Ubiquitin protein ligase binding	6/45	298/18,410	8.19 e-05	7.02 e-03	4.81 e-03
MF	GO: 0044389	Ubiquitin-like protein ligase binding	6/45	317/18,410	1.15 e-04	7.02 e-03	4.81 e-03
MF	GO: 0042277	Peptide binding	6/45	322/18,410	1.25 e-04	7.02 e-03	4.81 e-03
MF	GO: 0005048	Signal sequence binding	3/45	50/18,410	2.47 e-04	9.40 e-03	6.45 e-03
KEGG	hsa05131	Shigellosis	9/35	247/8,164	6.52 e-07	6.88 e-05	4.25 e-05
KEGG	hsa04621	NOD-like receptor signaling pathway	8/35	184/8,164	8.00 e-07	6.88 e-05	4.25 e-05
KEGG	hsa04722	Neurotrophin signaling pathway	6/35	119/8,164	9.71 e-06	5.57 e-04	3.44 e-04
KEGG	hsa04210	Apoptosis	6/35	136/8,164	2.09 e-05	8.98 e-04	5.55 e-04
KEGG	hsa05417	Lipid and atherosclerosis	7/35	215/8,164	2.86 e-05	9.84 e-04	6.08 e-04

GO, gene ontology; BP, biological process; CC, cellular component; MF, molecular function; KEGG, kyoto encyclopedia of genes and genomes; PRDEGs, pyroptosis-related differentially expressed genes.

### 3.5 GSEA

**FIGURE 5:**
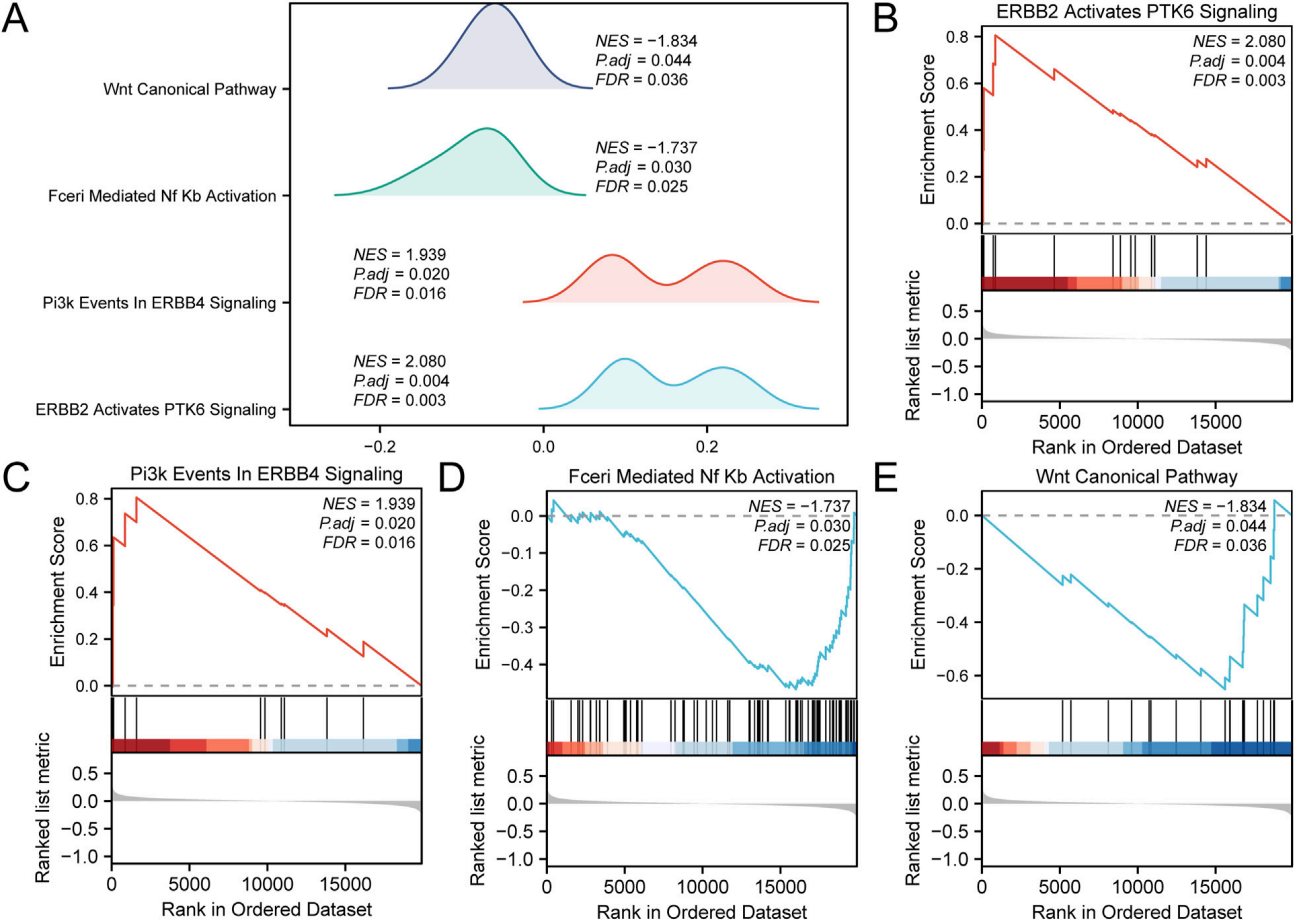
Gene set enrichment analysis (GSEA) for combined datasets **(A)** Results of the GSEA for the combined datasets, presented as ridge plots illustrating four biological functions, each including a NES, adjusted p-value (Padj), and FDR value, with results indicating the effect and statistical significance of each pathway. **(B)** The plot for “ERBB2 Activates PTK6 Signaling,” showing the relationship between gene rankings in the ordered dataset and the enrichment score. **(C)** The plot for “Pi3K Events in ERBB4 Signaling,” displaying the performance of genes within the ordered dataset. **(D)** The plot for “Fceri-Mediated Nf-κB Activation,” illustrating the variation in enrichment score with dataset ranking. **(E)** The plot for “Wnt Canonical Pathway,” showing the gene enrichment status of this pathway and its statistical analysis results. CA, Childhood Asthma; GSEA, Gene Set Enrichment Analysis; NES, normalized enrichment score. The screening criteria for GSEA were adj.p < 0.05 and FDR (q-value) < 0.25, with the p-value correction method being Benjamini-Hochberg (BH).

GSEA was performed to determine the conformity of the GEO dataset (combined datasets) of all gene expression levels in CA. This analysis evaluated the involvement of all expressed genes in BPs, CCs, and MFs (Figure 5A). Detailed results are presented in Table 3. The results showed that all genes in the combined datasets were significantly enriched in ERBB2, PTK6 signaling (Figure 5B), and Pi3K events in ERBB4 signaling (Figure 5C), biologically relevant functions, signaling pathways such as Fceri-mediated Nf-κB activation (Figure 5D), and the PID Wnt canonical pathway (Figure 5E).

**TABLE 3:** Results of gene set enrichment analysis (GSEA) for combined datasets.

ID	Set size	Enrichment score	NES	p-value	p-adjust	q-value
REACTOME_ERBB2_ACTIVATES_PTK6_SIGNALING	13	0.81	2.08	1.06 e-04	3.69 e-03	3.05 e-03
REACTOME_ERBB2_REGULATES_CELL_MOTILITY	14	0.78	2.05	3.43 e-04	8.86 e-03	7.30 e-03
REACTOME_INTERLEUKIN_2_SIGNALING	12	0.80	2.03	1.97 e-04	6.08 e-03	5.02 e-03
REACTOME_CLASS_C_3_METABOTROPIC_GLUTAMATE_PHEROMONE_RECEPTORS	36	0.62	2.02	1.00 e-04	3.53 e-03	2.91 e-03
REACTOME_IMMUNOREGULATORY_INTERACTIONS_BETWEEN_A_LYMPHOID_AND_A_NON_LYMPHOID_CELL	112	0.49	2.00	7.57 e-06	4.85 e-04	4.00 e-04
KEGG_TASTE_TRANSDUCTION	48	0.58	1.98	5.46 e-05	2.25 e-03	1.86 e-03
KEGG_NATURAL_KILLER_CELL_MEDIATED_CYTOTOXICITY	107	0.49	1.97	1.59 e-05	8.53 e-04	7.03 e-04
WP_EICOSANOID_SYNTHESIS	19	0.69	1.95	1.23 e-03	2.07 e-02	1.70 e-02
REACTOME_PI3K_EVENTS_IN_ERBB4_SIGNALING	10	0.80	1.94	1.14 e-03	2.00 e-02	1.65 e-02
WP_CHOLESTEROL_SYNTHESIS_DISORDERS	18	0.68	1.91	2.23 e-03	3.09 e-02	2.55 e-02
PID_ERBB_NETWORK_PATHWAY	15	0.70	1.86	2.41 e-03	3.28 e-02	2.71 e-02
REACTOME_CHEMOKINE_RECEPTORS_BIND_CHEMOKINES	55	0.52	1.85	3.49 e-04	8.86 e-03	7.30 e-03
REACTOME_SYNTHESIS_OF_PROSTAGLANDINS_PG_AND_THROMBOXANES_TX	13	0.71	1.84	3.44 e-03	4.24 e-02	3.49 e-02
KEGG_AUTOIMMUNE_THYROID_DISEASE	33	0.57	1.82	2.05 e-03	3.00 e-02	2.47 e-02
REACTOME_DAP12_INTERACTIONS	34	0.56	1.81	2.13 e-03	3.06 e-02	2.52 e-02
REACTOME_NUCLEAR_SIGNALING_BY_ERBB4	30	0.57	1.80	2.40 e-03	3.28 e-02	2.70 e-02
KEGG_ALLOGRAFT_REJECTION	25	0.59	1.74	4.24 e-03	4.68 e-02	3.86 e-02
REACTOME_SENSORY_PERCEPTION_OF_TASTE	44	0.51	1.72	3.22 e-03	4.02 e-02	3.32 e-02
REACTOME_FCERI_MEDIATED_NF_KB_ACTIVATION	77	0.47	1.74	2.05 e-03	3.00 e-02	2.47 e-02
PID_WNT_CANONICAL_PATHWAY	20	0.65	1.83	3.59 e-03	4.38 e-02	3.61 e-02

GSEA, gene set enrichment analysis; NES, normalized enrichment score.

### 3.6 Construction of PPI networks and screening of hub genes

**FIGURE 6:**
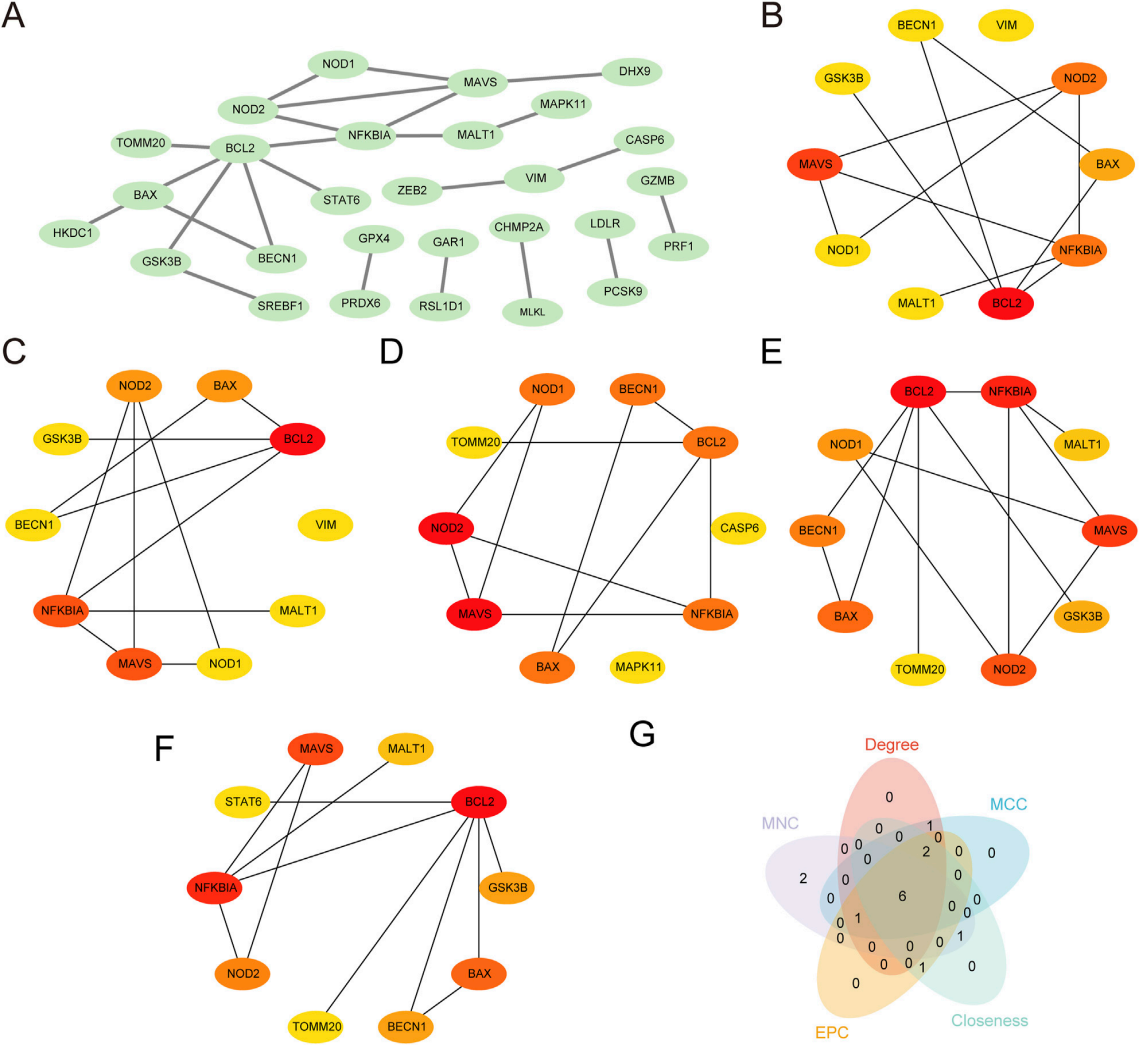
Protein–protein interaction (PPI) network and hub genes analysis **(A)** PPI network of PRDEGs constructed using the STRING database, displaying only protein nodes with interaction relationships. **(B–F)** PPI networks of the top 10 PRDEGs calculated using the cytoHubba plugin in Cytoscape software based on the following five algorithms: **(B)** Maximal Clique Centrality (MCC), **(C)** Maximum Neighborhood Component (MNC), **(D)** Degree, **(E)** edge-percolated component (EPC), and **(F)** Closeness. In each plot, circle colors range from red to yellow, indicating scores from high to low. **(G)** Venn diagram of the top 10 PRDEGs identified by the five algorithms, showing the intersection of hub genes; based on the intersection results, the identified CA hub genes include *NOD2*, *NFKBIA*, *BAX, BECN1, MAVS,* and *BCL2*. CA, Childhood Asthma; PPI, protein-protein interaction; PRDEGs, pyroptosis-related differentially expressed genes.

PPI analysis of 45 PRDEGs was performed using the STRING database to construct a PPI network (Figure 6A). Among these, 28 PRDEGs exhibited significant interactions (Supplementary Table S3). The following five algorithms from the cytoHubba plugin in Cytoscape were subsequently applied to evaluate the network properties of the 28 PRDEGs: MCC, degree, MNC, EPC, and closeness. The top 10 PRDEGs identified by each algorithm were visualized in their respective PPI networks as follows: MCC (Figure 6B), MNC (Figure 6C), degree (Figure 6D), EPC (Figure 6E), and closeness (Figure 6F). The nodes in these networks were color-coded from red to yellow to represent scores from high to low. Finally, intersecting top genes from all algorithms were displayed in a Venn diagram (Figure 6G), identifying the following six hub genes associated with CA: *NOD2, NFKBIA, BAX, BECN1, MAVS*, and *BCL2*.

### 3.7 Construction of regulatory network

**FIGURE 7:**
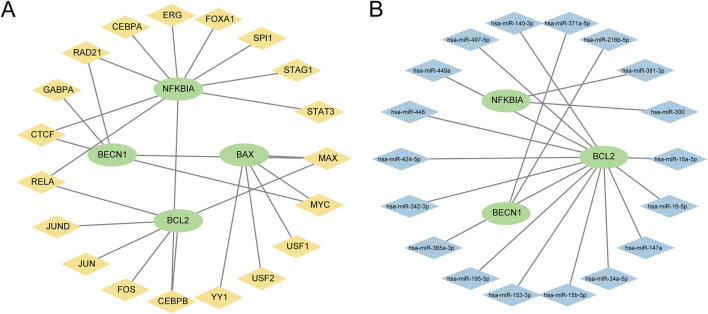
Regulatory network of pyroptosis-related differential genes (PRDEGs) **(A)** The mRNA-TF regulatory network of PRDEGs, illustrating the interaction relationships between 4 PRDEGs and 19 TFs; in the network, green nodes represent mRNAs, and yellow nodes represent TFs. **(B)** mRNA-miRNA regulatory network of PRDEGs, showing the interaction relationships between three PRDEGs and 18 miRNAs; in the network, green nodes represent mRNAs, and blue nodes represent miRNAs. PRDEGs, pyroptosis-related differentially expressed genes; TF, transcription factor.

First, TFs associated with PRDEGs were obtained from the hTF target database. These data were employed to construct and visualize the mRNA-TF regulatory network using Cytoscape software (Figure 7A). This network included four PRDEGs and 19 TFs (Supplementary Table S4). Similarly, miRNAs targeting PRDEGa were retrieved from the StarBase database to construct an mRNA-miRNA regulatory network visualized using Cytoscape software (Figure 7B). This network comprised three PRDEGs and 18 miRNAs (Supplementary Table S5).

### 3.8 Differential expression and ROC analysis of hub genes

**FIGURE 8:**
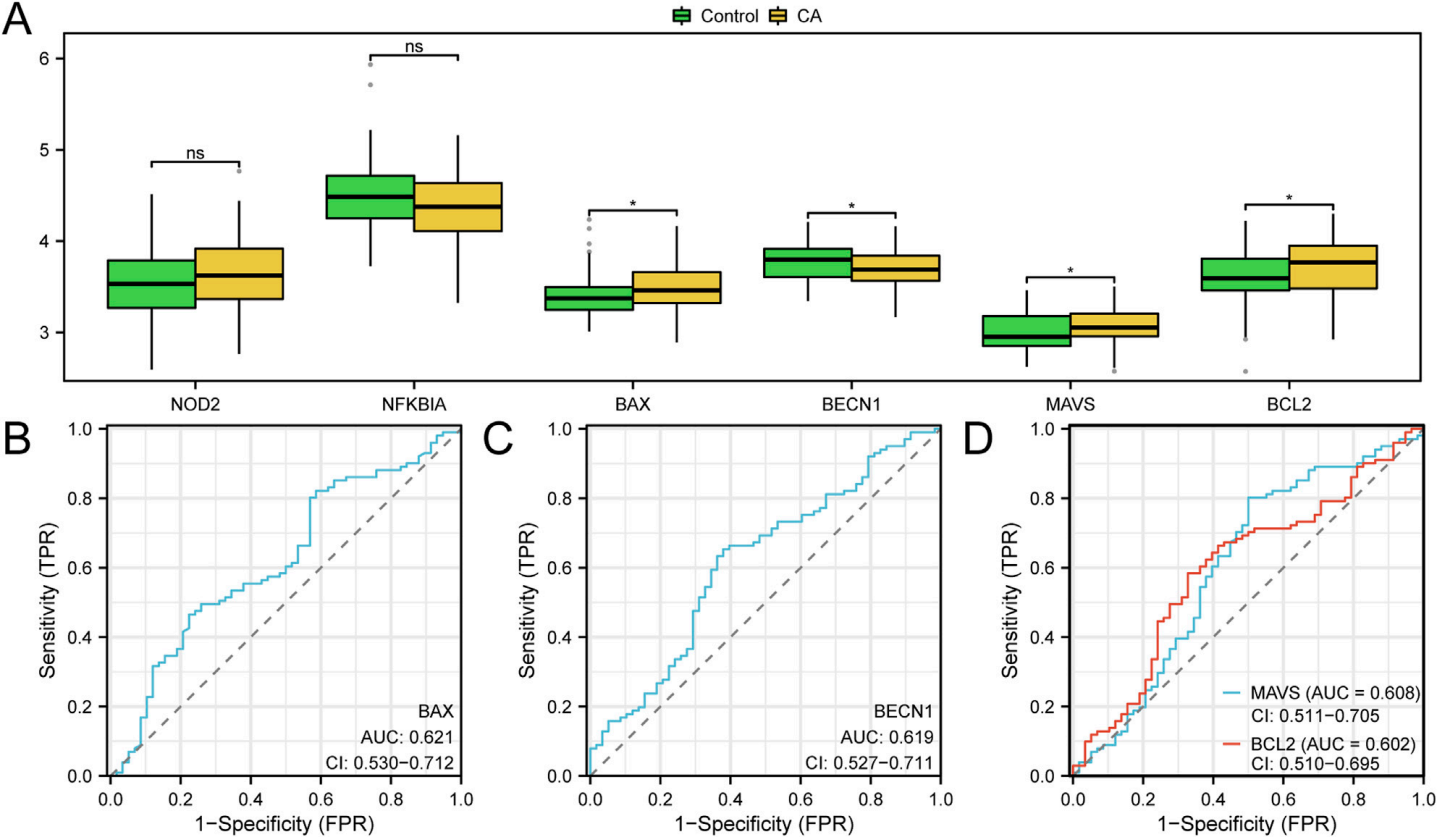
Differential expression validation and receiver operating characteristic (ROC) curve analysis **(A)** Group comparison plot of hub genes in the combined GEO datasets between the CA group and the control group, where green boxplots represent the control group and yellow boxplots represent the CA group; statistical analysis indicates significant expression differences for four hub genes (*BAX*, *BECN1*, *MAVS*, and *BCL2*) between the two groups (*p-value <0.05; ns: p-value ≥0.05, no statistical significance). **(B–D)** ROC curve analysis of hub genes, demonstrating the classification ability of genes *BAX* **(B)**, *BECN1* **(C)**, and *MAVS* and *BCL2* **(D)** in the combined GEO datasets (ns: no statistical significance; * p-value <0.05); when AUC >0.5, it indicates a trend toward promoting the event, with higher AUC values closer to 1 reflecting better diagnostic performance, while AUC values above 0.5 indicate low accuracy. Green represents the control group, and yellow represents the CA group. CA, childhood asthma; ROC, receiver operating characteristic; AUC, area under the curve; TPR, true positive rate; FPR, false positive rate.

To evaluate the expression of hub genes, a group comparison plot (Figure 8A) showed the expression levels of six hub genes between the CA and control groups within the combined GEO datasets. Differential expression analysis indicated that the expression of four hub genes—*BAX, BECN1, MAVS*, and *BCL2*—was significantly different between the CA and control groups (p < 0.05). ROC curves generated using the R package pROC assessed the diagnostic accuracy of the hub gene expression levels in distinguishing patients with CA from controls (Figures 8B–D). The ROC analysis indicated that *BAX, BECN1, MAVS*, and *BCL2* had moderate diagnostic accuracy (AUC >0.5) for distinguishing between CA and control samples.

### 3.9 Immune infiltration analysis of CA (CIBERSORT)

**FIGURE 9:**
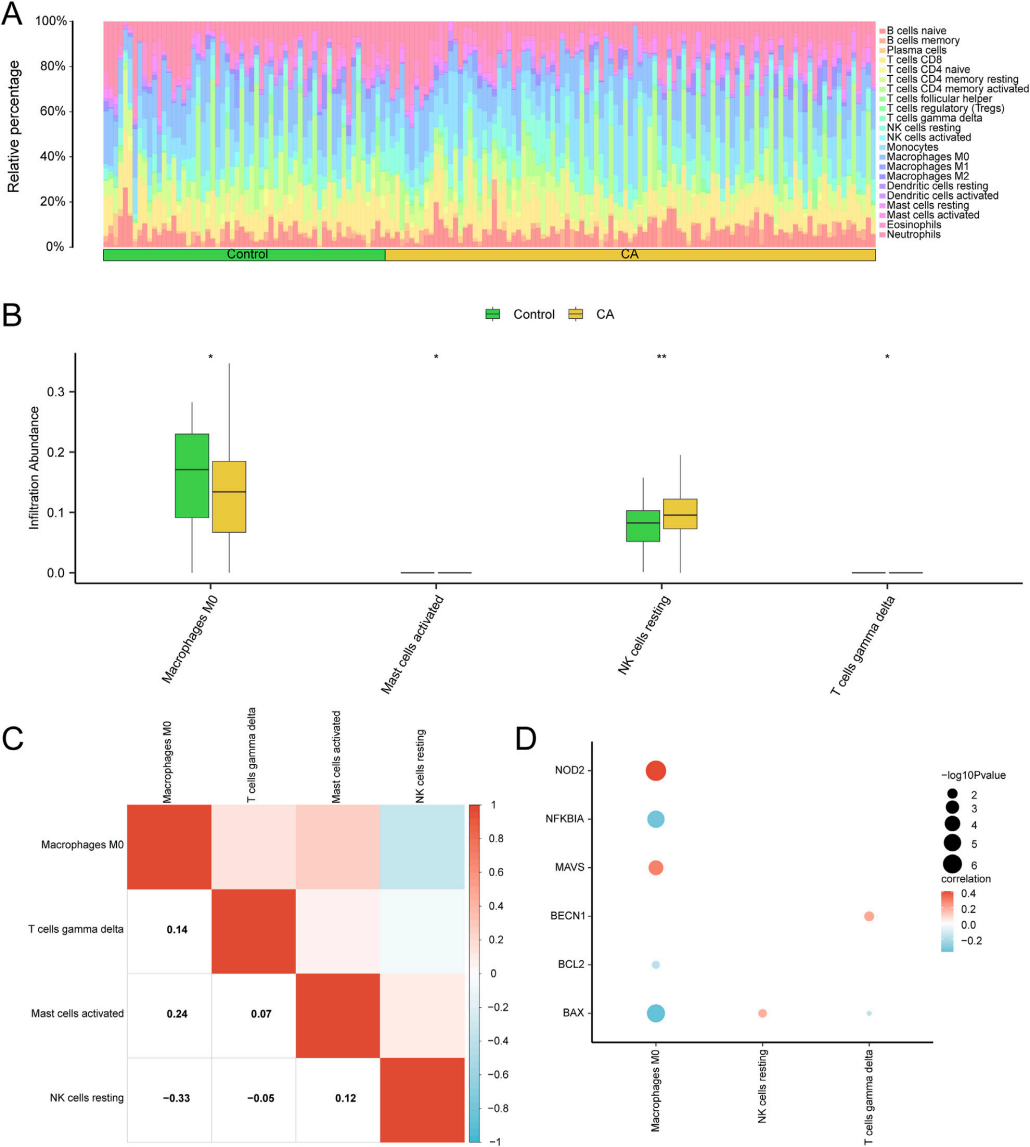
Combined datasets immune infiltration analysis using ssGSEA algorithm. Bar graph **(A)** and group comparison graph **(B)** show the proportion of immune cells in the childhood asthma (CA) and control groups. **(C)** Heatmap depicting the correlation between infiltrating abundance of immune cells with significant differences across the combined GEO datasets in group comparison plots. **(D)** Heatmap illustrating the correlation between pyroptosis-related hub genes (hub genes) and the infiltration abundance of seven immune cell types in the integrated GEO datasets (combined datasets). ssGSEA, single-sample gene-set enrichment analysis. * represents p value <0.05, statistically significant; ** represents p < 0.01 and highly statistically significant. Correlation coefficients (r-value) with absolute values below 0.3 were considered weak or irrelevant, while those between 0.3 and 0.5 indicated a weak correlation. Medium green grouping comparison chart for the control (control) group and yellow for the childhood asthma group (CA).

The CIBERSORT algorithm was used to calculate the abundance of 22 immune cell types in the combined GEO datasets. Immune infiltration analysis produced a bar chart showing the proportions of immune cells (Figure 9A), while group comparison plots (Figure 9B) highlighted differences in immune cell infiltration between the CA and control groups. Cell abundance (p < 0.05) differed significantly for macrophages M0, activated mast cells, and γδ T cells. Moreover, resting natural killer (NK) cells showed a highly significant difference (p < 0.01) between the two groups.

The correlation heatmap (Figure 9C) illustrated relationships among immune cell types. Macrophages M0 showed the strongest positive correlation with activated mast cells (r = 0.24) and the strongest negative correlation with resting NK cells (r = −0.33). Correlation bubble plots (Figure 9D) were used to visualize the association between hub genes and immune cell infiltration in the combined GEO datasets. A significant positive correlation was found between *NOD2* and macrophages M0 (r > 0.0, p < 0.05), and a significant negative correlation was observed between *BAX* and macrophages M0 (r < 0.0, p < 0.05).

### 3.10 Validation of Hub Gene Expression by qPCR

**FIGURE 10:**
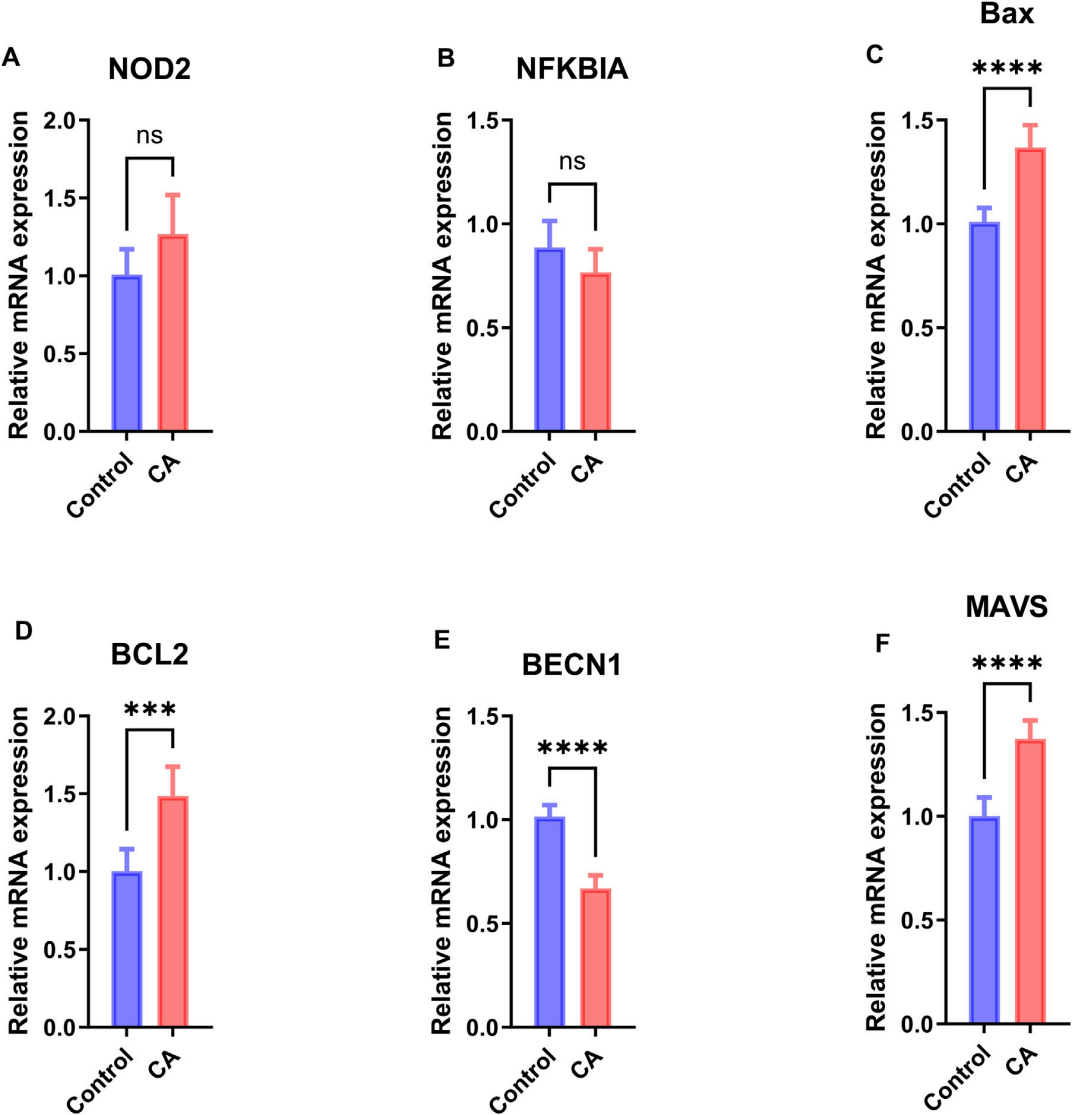
qPCR Validation of Hub Gene Expression. Relative mRNA expression levels of hub genes **(A)** NOD2, **(B)** NFKBIA, **(C)** BAX, **(D)** BCL2, **(E)** BECN1, and **(F)** MAVS in peripheral blood samples from 6 patients with childhood asthma (CA) and 6 healthy controls, measured by qPCR. Data are presented as mean ± standard deviation (SD). Statistical significance was determined by the two-sided unpaired Welch’s t-test, with significance levels indicated as follows: *p < 0.05, **p < 0.01, ***p < 0.001, ****p < 0.0001. Blue bars represent the control group, and red bars represent the CA group.

To validate the differential expression of hub genes identified through bioinformatics analysis, qPCR was performed on peripheral blood samples from six patients with CA and six healthy controls, revealing distinct mRNA expression patterns for the six hub genes (Figure 10). Specifically, *BAX*, *MAVS*, and *BCL2* exhibited significantly increased expression in the CA group (p < 0.0001 for *BAX* and *MAVS*, p < 0.001 for *BCL2*), while *BECN1* showed significantly decreased expression (p < 0.0001). In contrast, *NOD2* and *NFKBIA* showed no significant changes in expression.

## 4 Discussion

The pathogenesis of CA is multifaceted, involving intricate interactions among genetic, environmental, and immune-mediated mechanisms, which underscores the need to identify molecular pathways driving disease-associated inflammation. Through integrative analysis of GEO datasets, this study identified 2,069 DEGs, comprising 1,158 upregulated and 911 downregulated genes in CA samples compared with controls, highlighting the complex genetic landscape of asthma. Among these, 45 PRDEGs significantly enriched in immune response pathways such as the NOD-like receptor signaling pathway and apoptosis were identified (Table 2). These results emphasize the critical role of pyroptosis in CA inflammation, aligning with prior studies that link pyroptosis to airway hyperreactivity and inflammatory responses (Mihailidou et al., 2004; Szefler, 2007; Zhuang et al., 2020). Notably, hub genes such as BECN1 and BCL2, associated with cell death and inflammatory pathways in our analyses, are primarily linked to autophagy in existing literature, with their direct roles in canonical pyroptosis pathways remaining unclear. Recent studies suggest complex interactions between autophagy and programmed cell death, including pyroptosis and apoptosis, potentially mediated by inflammasome modulation or stress responses (Lin et al., 2024; Mao et al., 2024). These interactions require further experimental validation. Thus, we interpret these genes as potential bridges across multiple cell death and inflammatory processes, rather than core pyroptosis factors. Future studies should elucidate their dynamic regulation between autophagy and pyroptosis to clarify their impact on asthma pathogenesis.Dysregulation of these pathways likely contributes to chronic inflammation in CA, offering potential therapeutic targets for modulating inflammatory cell death pathways (Xu et al., 2023). Future research should explore the interactions between PRDEGs and classical asthma-related signaling pathways to enable the development of targeted therapies (Zar and Levin, 2012; Castagnoli et al., 2023).

Immune cell infiltration analysis using the CIBERSORT algorithm revealed significant differences between the CA and control groups, with increased abundance of macrophages M0, activated mast cells, and γδ T cells, and decreased resting NK cells in the CA group (Figure 9B). These findings suggest that specific immune mediators contribute to CA pathogenesis, with activated mast cells potentially driving allergic responses and macrophages M0 playing a dual role in promoting and resolving inflammation (Goretzki et al., 2021; Melgaard et al., 2024). Notably, macrophages M0 were correlated positively with *NOD2* expression (r > 0.0, p < 0.05) and negatively with *BAX* (r < 0.0, p < 0.05) (Figure 9D), indicating that pyroptosis-related hub genes may modulate immune cell dynamics in CA. Understanding these interactions could inform targeted therapies aimed at rebalancing immune responses in pediatric asthma patients. Importantly, Li et al. employed single-cell RNA-seq technology to thoroughly investigate the dynamic changes in immune cells during bone marrow immune infiltration and inflammaging, offering valuable insights into the regulation of the immune microenvironment (Li et al., 2023). Although the backgrounds of the two studies are different, with Li et al. primarily focusing on bone marrow immunity and inflammaging and ours emphasizing the immune regulatory mechanisms in CA, their findings on how changes in the immune system affect chronic inflammatory diseases still hold certain reference value for our understanding of immune imbalance in asthma.

Among the six hub genes identified, *BAX, BECN1, MAVS,* and *BCL2* exhibited statistically significant differences in expression between the CA and control groups based on GEO data (p < 0.05, Figure 8A), with AUC values ranging from 0.602 to 0.621 (95% CI: 0.510–0.712, Figures 8B–D), suggesting their potential as diagnostic biomarkers. These findings align with previous studies linking these genes to inflammatory and cell death pathways in CA and other immune-related conditions. For instance, *BAX* and *BCL2*, key regulators of apoptosis, are implicated in airway remodeling and immune activation in CA: *BAX* promotes cell death, contributing to tissue damage and inflammation, while *BCL2* enhances cell survival, potentially supporting the persistence of immune cells in inflamed tissues (Vervliet et al., 2023). *MAVS*, crucial for antiviral responses, is associated with CA exacerbation, reflecting the role of viral infections in symptom severity (Rich et al., 2020). *BECN1,* involved in autophagy, may interact with pyroptosis to modulate immune responses, though its specific role in CA remains unclear (Chiok and Bose, 2022). qPCR validation in our small cohort confirmed significantly increased expression of *BAX, MAVS*, and *BCL2* in the CA group (p < 0.0001 for *BAX* and *MAVS*, p < 0.001 for BCL2), while *BECN1* showed significantly decreased expression (p < 0.0001; Figure 10). The decreased expression of *BECN1*, consistent with GEO data, suggests a potential impairment in autophagy, which may exacerbate inflammation by reducing its regulatory interaction with pyroptosis (Chiok and Bose, 2022). This finding warrants further investigation into the balance between autophagy and pyroptosis in CA pathogenesis. In contrast, *NOD2* and *NFKBIA* showed no significant differences in expression in either GEO data (Figure 8A) or qPCR validation (Figure 10). However, the positive correlation of *NOD2* with macrophages M0 (r > 0.0, p < 0.05; Figure 9D) suggests a potential role in modulating immune responses, warranting further exploration (Platnich and Muruve, 2019). Conversely, the expression pattern of NFKBIA, an inhibitor of NF-κB signaling, could contribute to unchecked inflammatory responses, aligning with the heightened immune activation observed in CA (Xu et al., 2023). Targeting hub genes with significant expression changes, such as *BAX, BECN1, MAVS*, and *BCL2,* could modulate inflammatory and cell death pathways, offering a personalized treatment approach for CA. Additionally, the role of *NOD2* in immune regulation makes it a potential therapeutic target despite showing no significant difference in expression.

Emerging research indicates that targeting pyroptosis-related genes can influence inflammatory responses and immune regulation, providing new directions for asthma treatment. For example, the NLRP3 inflammasome, a member of the NOD-like receptor family, is associated with CA, and its excessive activation exacerbates airway inflammation (Huang et al., 2023). Given the role of NOD2 in the NOD-like receptor signaling pathway and its correlation with immune cell infiltration, targeting NOD2-NLRP3 interactions with inhibitors like MCC950, which has shown anti-inflammatory effects in animal models, could mitigate CA inflammation. Similarly, inhibiting Gasdermin D, the terminal execution protein of pyroptosis, may reduce airway epithelial damage in patients with asthma (Wu et al., 2024). Additionally, PI3K/Akt/mTOR inhibitors have been explored in pulmonary inflammation and may modulate pyroptosis-related pathways, potentially alleviating the inflammatory state in CA (Pan et al., 2023). Elucidating the interplay between pyroptosis and classical inflammatory pathways may provide new insights into the multifaceted pathogenesis of CA, facilitating the development of more effective, personalized treatment modalities (Chen et al., 2024). Future studies should validate the diagnostic potential of hub genes with significant expression differences, such as *BAX, BECN1, MAVS,* and *BCL2*, using larger CA cohorts with diverse clinical phenotypes (e.g., allergic vs. non-allergic asthma) and explore their therapeutic implications through *in vitro* and *in vivo* models, such as CRISPR-based gene editing or animal models of CA, to pave the way for personalized treatment strategies. Additionally, the immune-regulatory role of *NOD2* warrants further investigation in the context of CA.

Despite comprehensive bioinformatics analysis and qPCR validation, this study has several limitations. First, the GEO dataset (101 CA cases, 58 controls) may not fully capture CA heterogeneity, and the small qPCR cohort (6 CA patients, 6 controls) limits statistical power, necessitating larger studies. Second, the absence of comprehensive clinical metadata (e.g., disease severity, FEV1, IgE levels) in GEO datasets (GSE27011, GSE40888) and incomplete pyroptosis pathway annotations in mainstream databases (e.g., KEGG, GO) restricted analyses of PRDEGs’ clinical associations, multi-factor machine learning modeling, and direct detection of pyroptosis-specific pathways. While NOD-like receptor signaling and apoptosis pathways are linked to pyroptosis, and CIBERSORT analyses suggest immune cell associations (e.g., *NOD2* with M0 macrophages), experimental validation of pyroptosis mechanisms and causal relationships is lacking. Future research will utilize larger independent cohorts with rich clinical metadata, leverage pyroptosis-specific databases, and apply machine learning to develop multi-factor models. Additionally, single-cell RNA sequencing and OVA-induced asthma models will validate pyroptosis-immune interactions and construct a “pyroptosis-immune-inflammation” regulatory network to clarify hub gene roles.

In summary, this study provides novel insights into the role of PRGs in CA. By integrating and analyzing data from multiple GEO datasets, we identified 45 PRDEGs and constructed a comprehensive regulatory network involving miRNAs and TFs. The hub genes, particularly *BAX, BECN1, MAVS*, and *BCL2*, demonstrated significant differential expression and moderate diagnostic potential. Immune infiltration analysis revealed increased macrophages M0, activated mast cells, and γδ T cells in CA, with a positive correlation between *NOD2* and macrophages M0 (Figure 9D), highlighting potential interactions between PRGs and the immune system. These findings offer promising avenues for future research into the diagnostic and therapeutic applications of pyroptosis-related pathways in CA management.

## Data Availability

The datasets presented in this study can be found in online repositories. The names of the repository/repositories and accession number(s) can be found in the article/Supplementary Material.

## Abbreviations

CA, childhood asthma; DEGs, differentially expressed genes; PRGs, pyroptosis-related genes; PRDEGs, pyroptosis-related differentially expressed genes; GSEA, gene set enrichment analysis; GO, gene ontology; KEGG, Kyoto Encyclopedia Of Genes And Genomes; PPI, protein–protein interaction; ROC curve, receiver operating characteristic curve; BH, Benjamini–Hochberg; BP, biological process; CC, cellular component; MF, molecular function; GEO, Gene Expression Omnibus; PCA, principal component analysis; MCC, maximal clique centrality; MNC, maximum neighborhood component; EPC, edge-percolated component; TF, transcription factor; AUC, area under the curve; NK cells, natural killer cells.
